# Mitochondrial control of hypoxia-induced pathological retinal angiogenesis

**DOI:** 10.1007/s10456-024-09940-w

**Published:** 2024-08-03

**Authors:** Hitomi Yagi, Myriam Boeck, Shen Nian, Katherine Neilsen, Chaomei Wang, Jeff Lee, Yan Zeng, Matthew Grumbine, Ian R. Sweet, Taku Kasai, Kazuno Negishi, Sasha A. Singh, Masanori Aikawa, Ann Hellström, Lois E. H. Smith, Zhongjie Fu

**Affiliations:** 1 Department of Ophthalmology, Boston Children’s Hospital, Harvard Medical School, 3 Blackfan Circle, CLS 18, Boston, MA 02115 USA; 2 Department of Ophthalmology, Keio University School of Medicine, Tokyo, 160-8582 Japan; 3 Eye Center, Medical Center, Faculty of Medicine, University of Freiburg, 79106 Freiburg, Germany; 4 Department of Pathology, Xi’an Medical University, Xi’an, 710021 Shaanxi Province China; 5 EnTox Sciences, Inc, Mercer Island, WA 98040 USA; 6 University of Washington Medicine Diabetes Institute, University of Washington, Seattle, WA 98109 USA; 7 Center for Interdisciplinary Cardiovascular Sciences, Division of Cardiovascular Medicine, Department of Medicine, Brigham Women’s Hospital, Harvard Medical School, Boston, MA 02115 USA; 8 Center for Excellence in Vascular Biology, Division of Cardiovascular Medicine, Brigham and Women’s Hospital, Harvard Medical School, Boston, MA 02115 USA; 9 Channing Division of Network Medicine, Department of Medicine, Brigham Women’s Hospital, Harvard Medical School, Boston, MA 02115 USA; 10 The Sahlgrenska Centre for Pediatric Ophthalmology Research, Department of Clinical Neuroscience, Institute of Neuroscience and Physiology, Sahlgrenska Academy, University of Gothenburg, 405 30 Gothenburg, Sweden

**Keywords:** Retinal angiogenesis, Oxygen-induced retinopathy, Neovascularization, Mitochondrial respiration, Hypoxia, Retinopathy of prematurity

## Abstract

**Objective:**

Pathological retinal neovascularization is vision-threatening. In mouse oxygen-induced retinopathy (OIR) we sought to define mitochondrial respiration changes longitudinally during hyperoxia-induced vessel loss and hypoxia-induced neovascularization, and to test interventions addressing those changes to prevent neovascularization.

**Methods:**

OIR was induced in C57BL/6J mice and retinal vasculature was examined at maximum neovessel formation. We assessed total proteome changes and the ratio of mitochondrial to nuclear DNA copy numbers (mtDNA/nDNA) of OIR vs. control retinas, and mitochondrial oxygen consumption rates (OCR) in ex vivo OIR vs. control retinas (BaroFuse). Pyruvate vs. vehicle control was supplemented to OIR mice either prior to or during neovessel formation.

**Results:**

In OIR vs. control retinas, global proteomics showed decreased retinal mitochondrial respiration at peak neovascularization. OCR and mtDNA/nDNA were also decreased at peak neovascularization suggesting impaired mitochondrial respiration. In vivo pyruvate administration during but not prior to neovessel formation (in line with mitochondrial activity time course) suppressed NV.

**Conclusions:**

Mitochondrial energetics were suppressed during retinal NV in OIR. Appropriately timed supplementation of pyruvate may be a novel approach in neovascular retinal diseases.

**Supplementary Information:**

The online version contains supplementary material available at 10.1007/s10456-024-09940-w.

## Introduction

Local hypoxia and nutrient deprivation are among the important factors triggering retinal neovascularization, occurring after vascular loss [1–3]. Fragile neovessels can cause blindness in diabetic retinopathy and retinopathy of prematurity. Current treatments have adverse effects [4, 5]. Exploration of molecular mechanisms underlying the disease and its progression could lead to effective and safe therapeutic therapies.

Retinas exhibit a high metabolic demand [6]. Disruption of metabolic pathways results in retinal neural and vascular defects [7–9]. Mitochondrial respiration is required for energy production and retinal health [10–14]. Little is known about potential metabolic contribution to hypoxia-induced retinopathy. In mice with subretinal neovascularization, disturbed glucose and lipid supply to the retina disrupt mitochondrial respiration, leading to energy shortage and compensatory but pathological neovascularization [15]. Here, we examined mitochondrial changes during oxygen-induced retinopathy (OIR), a well-established model of retinal vessel loss and hypoxia-induced neovascularization [16].

In OIR, neonatal mice are exposed to hyperoxia from postnatal day (P)7-P12, causing retinal vessel loss and cessation of vessel growth, and returned to room air from P12-P17. Hypoxia and nutrient shortage in avascular retinal areas trigger the release of pro-angiogenic signals (such as vascular endothelial growth factor) and drive neovascularization to compensate for the lack of oxygen and nutrients. We used global retinal proteomics to assess major pathways affected during OIR. We further validated the identified pathways ex vivo and tested pyruvate supplementation as an intervention in OIR.

## Methods

Detailed experimental procedures are available in the Supplemental Methods.

## Results

### Decreased abundance of proteins involved in mitochondrial respiration in retinas with hypoxia-induced neovascularization (P17)

**Fig. 1:**
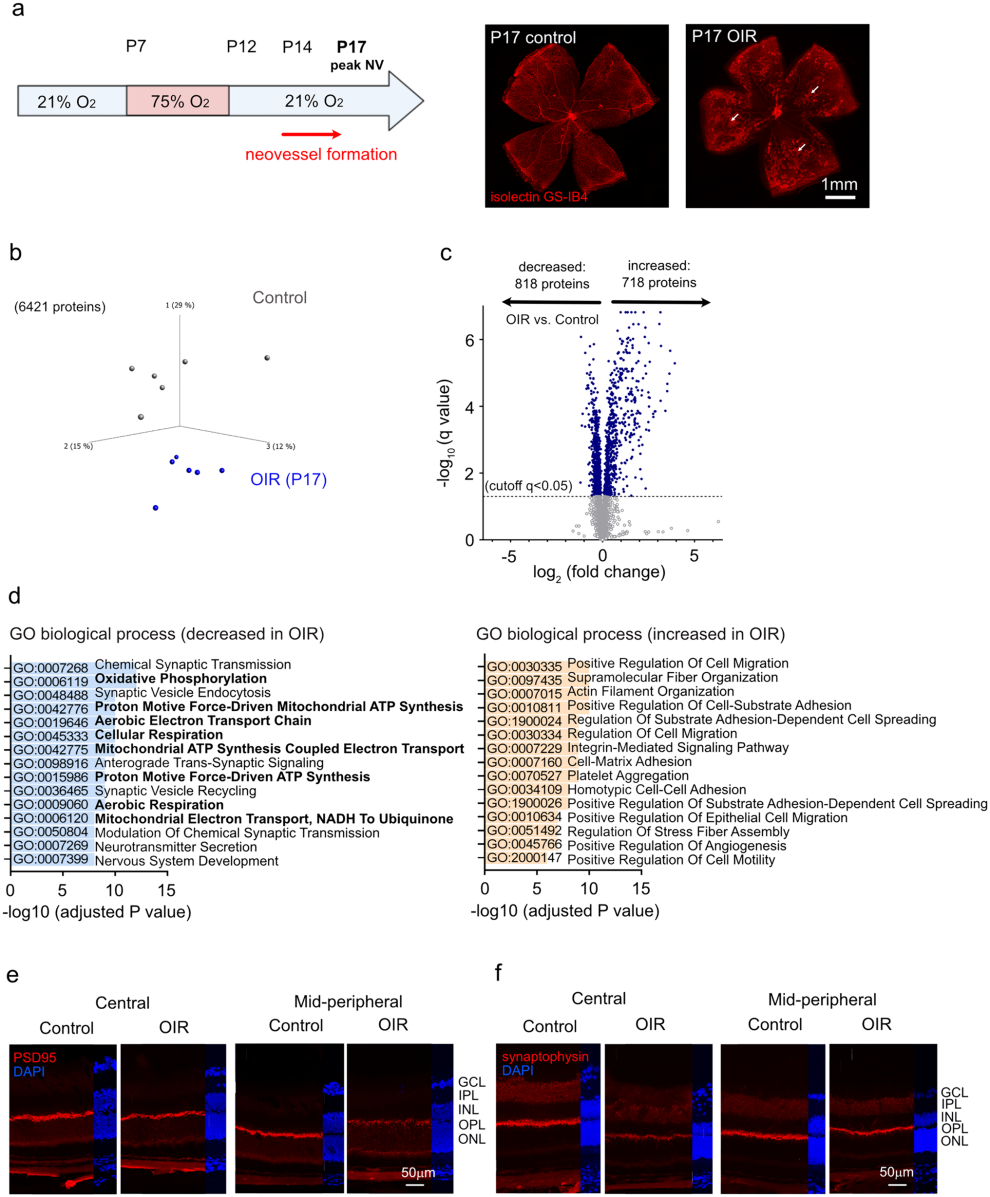
Global proteomic analysis of OIR vs. control mouse retinas. **a** Schematic of the mouse OIR model (left). Neovessels form from postnatal day (P)14 and peak at P17. Representative images of retinal whole mounts (right) at P17 with isolectin GS-IB4 vessel staining (red). OIR retinas exhibited central vaso-obliteration (VO) and mid-peripheral neovascularization (NV, white arrows). Scale bar 1 mm. **b** Unfiltered principal component analysis of 6421 proteins depicting differences between retinal protein profiles of P17 OIR (blue) vs. control mice (grey). Each dot represents one retina (n = 6 retinas/group). **c** Volcano plot of differentially abundant proteins between P17 OIR vs. control retinas. Each data point represents a unique protein based on log_2_ (fold change) on the x-axis and -log_10_ (q value) on the y-axis. Cutoff corresponds to a false discovery rate-adjusted P value (q value) < 0.05. **d** Top-ranked Gene Ontology (GO) terms of biological processes with decreased (left) or increased (right) protein abundance (q < 0.05) in P17 OIR vs. control retinas. Pathways were sorted by -log_10_ (adjusted P value). Pathways for mitochondrial activity are highlighted in bold. **e**, **f** Immunostaining for retinal synapses in P17 OIR vs. control retinas: PSD95 (postsynaptic, left, red) and synaptophysin (presynaptic, right, red). Cell nuclei were labeled with 4′,6-diamidine-2′-phenylindole dihydrochloride (DAPI, blue). Images were taken in central and mid-peripheral retinal areas. *GCL* Ganglion cell layer, *IPL* inner plexiform layer, *INL* inner nuclear layer, *OPL* outer plexiform layer, *ONL* outer nuclear layer. Scale bar 50 µm

In OIR mice, relative hypoxia-induced mid-peripheral neovascularization starts at P14 and peaks at P17 [16] (Fig. 1a). To evaluate molecular changes in neovascular retinas at peak neovascularization, we conducted global proteomics of OIR and control mouse retinas at P17. Principal component analysis of unfiltered proteome revealed distinct protein profiles in OIR vs. control retinas (Fig. 1b) further depicted in a volcano plot (Fig. 1c). A total of 7870 proteins were identified, and 6421 proteins were characterized by at least 2 distinct unique peptides. Significant changes in abundance (q < 0.05) were observed in 1536 proteins. OIR retinas showed increased abundance of hypoxia-inducible factor 1 alpha (HIF1α) compared to control retinas (fold change 1.47, q = 0.02), in line with previously reported increased HIF1α stabilization in OIR retinas [17]. Gene ontology (GO) analysis of proteins with reduced abundance in OIR retinas uncovered decreased biological processes associated with mitochondrial respiration and synaptic vesicles (Fig. 1d). Proteins with increased abundance in OIR retinas exhibited GO enrichment involved in cell migration, platelet aggregation, and angiogenesis. We confirmed synaptic changes in central and mid-peripheral OIR vs. control retinas via immunohistochemistry using the synaptic markers synaptophysin and postsynaptic density protein 95 (PSD95) (Fig. 1e, f). In P17 OIR retinas, decreased synaptic staining was found in the outer plexiform layer where photoreceptors connect with their downstream inner neurons, confirming the reliability of proteomics analysis.

#### Decreased mitochondrial activity in neovascular retinas

To further validate mitochondrial dysfunction in P17 OIR retinas as suggested by proteomics GO analysis, mitochondrial to nuclear DNA copy number ratio (mtDNA/nDNA) and oxygen consumption rate (OCR) were examined in P12, P14, and P17 OIR vs. control retinas.

**Fig. 2:**
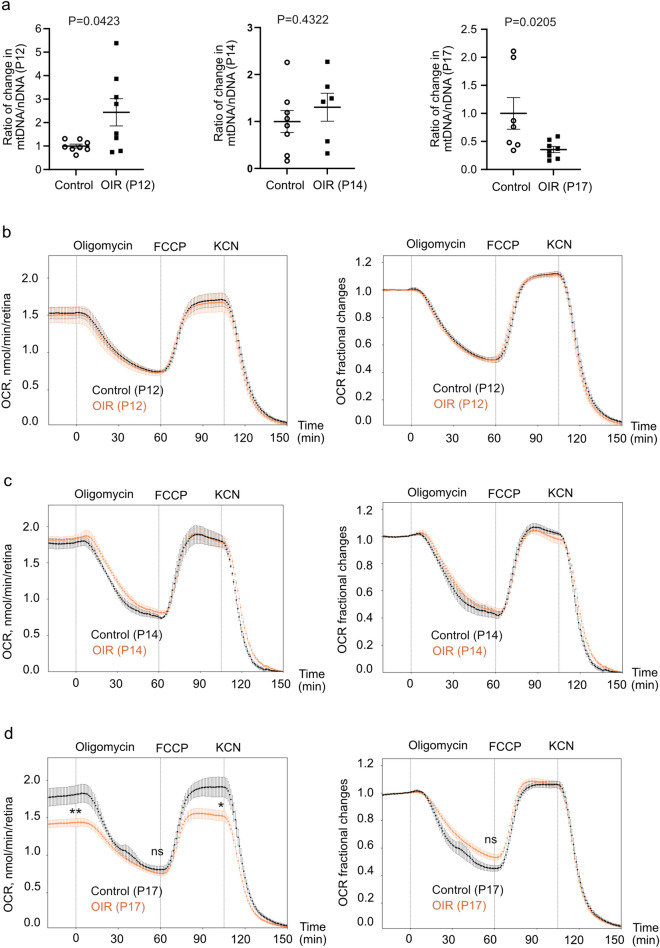
Impaired mitochondrial respiration in OIR vs. control retinas. **a** Altered mtDNA/nDNA in P12, P14, and P17 OIR vs. control retinas. Ratio of change was calculated and compared with control retinas. Unpaired t-test or Mann-Whitney test (n = 6-8 mice/group). **b**–**d** Oxygen consumption rate (OCR, left) and factional changes of OCR with baseline set to 1 (right) in P12 (n = 6 retinas/group), P14 (n = 3/group), and P17 (OIR, n = 16; control, n = 5) OIR and control retinas. Oligomycin A (ATP synthase inhibitor), carbonyl cyanide 4-(trifluoromethoxy)phenylhydrazone (FCCP, uncoupler of mitochondrial respiration), and potassium cyanide (KCN, complex IV inhibitor) were sequentially applied. Decreased OCR in P17 OIR vs. control retinas at baseline and at FCCP-induced maximal respiratory capacity confirmed by unpaired t-test. P < 0.01 (**), P < 0.05 (*), *ns* not significant

As mitochondrial DNA is highly susceptible to oxidative damage [18], oxygen exposure in OIR might result in mitochondrial impairment. However, in OIR vs. control retinas, mtDNA/nDNA ratio was increased at P12 after hyperoxia, unchanged at P14 and decreased at P17 (Fig. 2a).

We further assessed OCR reflecting mitochondrial respiration in P12, P14, and P17 OIR vs. control retinas in the presence of constant glucose (5 mM) and oxygen (21%). At P12 and P14, there were no significant changes in OCR between OIR and control retinas (Fig. 2b, c). At P17 OIR vs. control, total OCR was decreased during baseline condition and at maximal OCR reflected by the response to mitochondrial uncoupler carbonyl cyanide 4-(trifluoromethoxy)phenylhydrazone (FCCP) (Fig. 2d). Despite their diminished remaining respiratory capacity, P17 OIR retinas appeared energetically similar to control retinas as shown by the data graphed as a fraction of each group’s baseline OCR (Fig. 2d). The data did however suggest a trend towards increased uncoupling from ATP synthesis in OIR retinas as the OCR-decreasing effect of ATP synthase inhibitor oligomycin as a fraction of baseline was smaller in OIR vs. control retinas. In all groups, retinal OCR was partly uncoupled from ATP synthesis, and baseline OCR was close to maximal respiratory capacity, representing typical features of the retina [19].

#### Timed supplementation of pyruvate suppressed pathological retinal angiogenesis

**Fig. 3:**
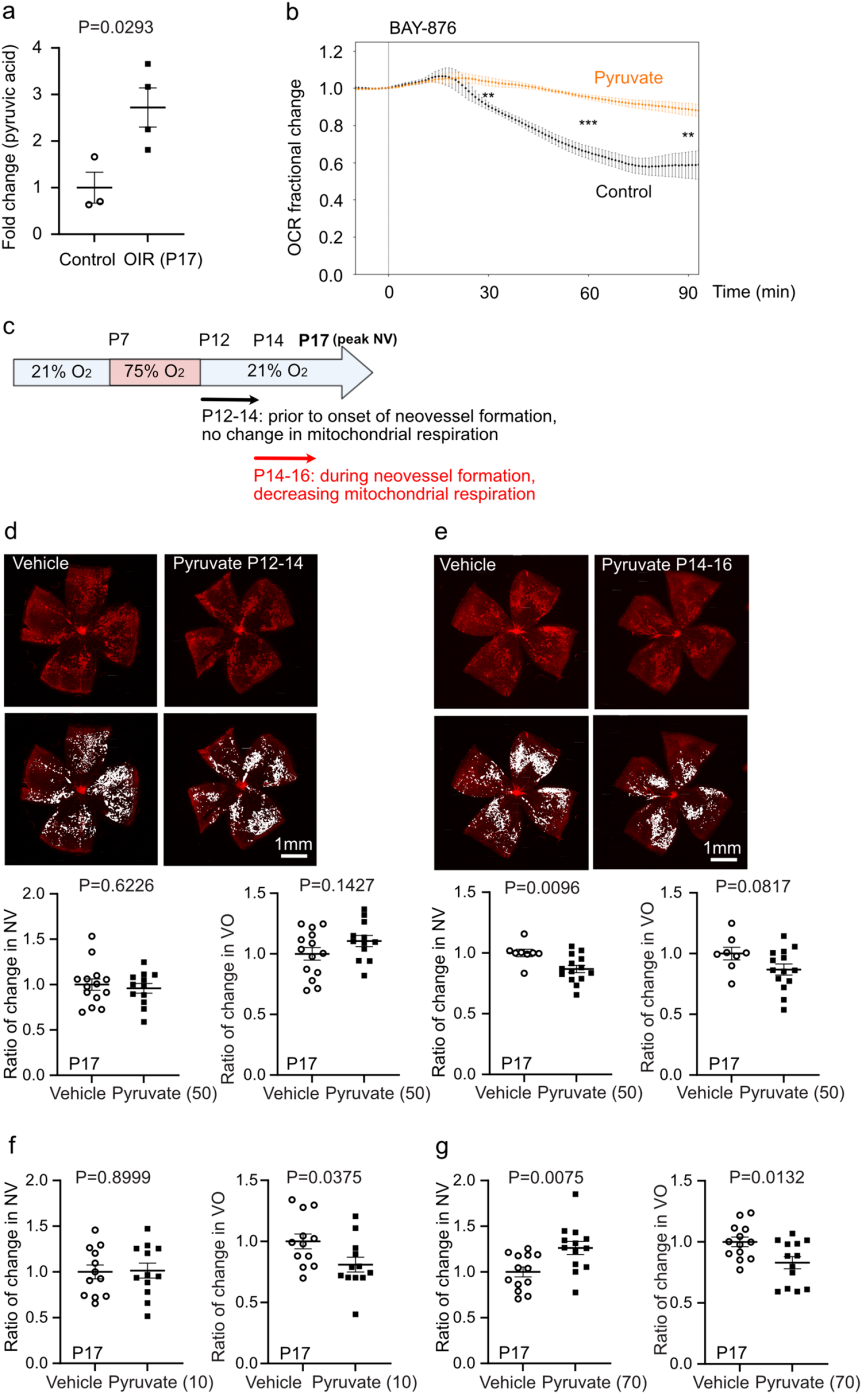
Pyruvate protected against hypoxia-induced pathological retinal angiogenesis. **a** Replot of pyruvic acid in OIR vs. control retinas at P17 from our prior publication. Unpaired t-test (n = 3-4 samples/group with 6 retinas/sample). (Tomita et al., Diabetologia. 2021 Jan;64(1):70–82). **b** Pyruvate sustained mitochondrial respiration under glucose deprivation conditions in OIR retinas. BaroFuse analysis of OCR in P17 C57BL/6J OIR retinas perfused with Krebs-Ringer Solution supplemented with 1 mM glucose only (control) vs. 1 mM glucose and 10 mM pyruvate. BAY-876 (glucose transporter inhibitor, 20 µM) was injected into both media to block glucose entry into the cells. Unpaired t-test was applied to detect differences in OCR 30, 60, and 90 min after BAY-876 injection between retinas treated with pyruvate vs. control (n = 4-5 retinas/group, 2 independent experiments). **P < 0.01, ***P < 0.001. **c** Schematics of pyruvate supplementation in OIR mice. Mouse pups were intraperitoneally injected with 50 µg/g pyruvate or vehicle daily either prior to or during neovessel formation. **d**, **e** At P17, neovascular (NV) and vaso-obliterated (VO) area were examined after 50 µg/g pyruvate or vehicle treatment from P12-P14 **d** or P14-P16 **e**. Scale bar 1 mm. Ratio of change was calculated and compared with vehicle group. Unpaired t-test (n = 8-14 retinas/group). **f**, **g** Quantification of NV, VO in OIR pups after 10 µg/g **f** or 70 µg/g **g** pyruvate vs. vehicle treatment from P14-P16. Unpaired t-test (n = 12-13 retinas/group)

Metabolomics analysis of P17 OIR vs. control retinas from our previous study [20] showed higher levels of pyruvate in P17 OIR retinas (Fig. 3a). To prove the concept that OIR retinas could uptake exogenous pyruvate for use as mitochondrial fuel, we isolated P17 OIR retinas and incubated them in medium with/without pyruvate. We found that OCR was preserved in pyruvate-supplemented medium after pharmaceutically blocking glucose uptake using BAY-876 (Fig. 3b). Whether pyruvate, potentially fueling mitochondria after conversion to acetyl-CoA, improves or worsens neovascularization is unclear. We administered pyruvate or vehicle control to littermate OIR mice from P12-P14 or P14-P16 (Fig. 3c). Pyruvate supplemented (50 µg/g) from P12-P14 prior to neovessel formation (during undiminished mitochondrial respiration) had no impact on P17 neovascularization (Fig. 3d). Pyruvate treatment (50 µg/g) during neovessel formation from P14-P16 (during switch to suppressed mitochondrial respiration) decreased P17 neovascularization (Fig. 3e). No differences in retinal vaso-obliteration or body weight were observed. Further assessment of pyruvate doses showed that a lower dose (10 µg/g, P14-P16) suppressed vaso-obliteration and had no impact on neovascularization (Fig. 3f). However, a higher dose (70 µg/g, P14-P16) suppressed vaso-obliteration and exacerbated neovascularization (Fig. 3g), suggesting that pyruvate protection against OIR was time- and dose-dependent.

## Discussion

Oxygen and nutrient deprivation drive the formation of pathological retinal neovessels. Our data showed a significant decrease in mitochondrial respiration in mouse OIR retinas between P14 and maximum neovessel proliferation at P17 during a period of poor normal vessel coverage. Supplementing pyruvate from P14-P16 during relative hypoxia and decreasing mitochondrial function suppressed the formation of retinal neovessels at P17, suggesting that providing metabolic substrates when mitochondrial function is suppressed might help control retinal neovascularization.

Regulating glucose metabolism and fatty acid oxidation can potentially modify physiological and pathological retinal angiogenesis. In human retinal endothelial cells in vitro, hypoxia decreased basal, ATP-linked, and maximal OCR [21]. In OIR, genetic attenuation of endothelial or microglial cell glycolysis suppresses retinal neovascularization [22–25]. De novo lipogenesis is required to maintain endothelial cell function and vascular sprouting [26]. Endothelial cell loss of carnitine palmitoyltransferase 1a, which transports long-chain fatty acids into mitochondria, impairs endothelial cell proliferation and compromises de novo nucleotide synthesis for DNA replication [27]. Endothelial cell loss of mitochondrial proteins (mitochondrial transcription factor A, respiratory complex IV component, or redox protein thioredoxin 2) retards vessel growth and pathological arteriovenous malformation in the developing retina [28]. In rat OIR, promoting in vivo glucose uptake by inhibiting mitochondrial uncoupling protein 2 during hyperoxia suppresses vaso-obliteration and late neovascularization [29]. During development, reduced glucose and lipid supply to photoreceptors triggers subretinal neovascularization in mice [6, 15]. These observations suggest a phase-specific, cell-specific, and substrate-specific metabolic contribution to retinal angiogenesis. We here showed that pyruvate supplementation during the onset of neovessel growth in OIR (P14-P16) associated with decreased mitochondrial copy number (mtDNA/nDNA) and function (OCR) reduced P17 neovascularization. Further investigations are required to elucidate the impact of metabolism on retinal vascular pathology.

Kim et al. assessed the proteome of P17 mouse OIR vs. control retinas [30] and found 38 differentially expressed proteins using the criteria P < 0.05, > 2 unique peptides, error factor < 2, and fold change > 1.2 or < 0.83. Proteins involved in “Glucose Metabolic Process” showed higher abundance and proteins involved in “Chemical Synaptic Transmission” had lower abundance in P17 OIR retinas. Here we also observed reduced abundance of proteins involved in the GO term “Chemical Synaptic Transmission” in P17 OIR retinas. Our proteomics dataset showed that in P17 OIR retinas, pathways involved in cell migration, platelet aggregation, and angiogenesis were increased. Pharmaceutical inhibition of endothelial cell migration suppresses retinal neovascularization in mouse OIR [31]. In preterm infants, platelet deficiency is associated with severe (treatment-requiring) proliferative retinopathy of prematurity [32]. In mouse OIR, platelet depletion during hypoxia (P15-P16) exacerbates vascular pathology, while platelet transfusions inhibit retinal neovascularization [32]. These reports reinforce the findings in our proteomics dataset and validate its capacity to reveal molecular mechanisms in retinal neovascularization. Recent transcriptomics analysis shows inconsistent changes in expression of genes involved in mitochondrial respiration in P17 OIR mouse retinas [33, 34]. However, with mitochondrial functional analysis (OCR) in ex vivo hypoxic P17 retinas with neovascularization, we found decreased mitochondrial respiration in line with decreased abundance of proteins involved in mitochondrial respiration identified with proteomics.

Our present work has limitations. We previously found several metabolites altered in OIR retinas [20]; however, only pyruvate was tested here, and whether pyruvate serves as a direct mitochondrial fuel in OIR needs to be validated in vivo. Also, the retinas were exposed to artificial oxygen and nutrient conditions in the Barofuse system, not fully reflecting in vivo retinas with much lower oxygen levels [35]. Although the mouse model of OIR has been employed to mimic hypoxia-induced neovascularization for almost three decades, it does not reflect all aspects of retinal neovessel formation.

## Conclusion

Mitochondrial function decreased during neovessel formation in hypoxic and insufficiently vascularized OIR retinas. Providing a metabolic intermediate, pyruvate, only during neovessel formation and mitochondrial dysfunction helped suppress retinal neovascularization. Our work highlights the importance of mitochondrial respiration in metabolic retinal disorders and suggests that timely supplementation of nutrients or metabolic intermediates may help prevent disease.

## Supplementary Information

Below is the link to the electronic supplementary material.

Supplementary file1 (DOCX 51 KB)

## Data Availability

All the data supporting the conclusions of this study are included within the article and supplementary data. All the other data and materials are available upon request to the corresponding author. The mass spectrometry proteomics data have been deposited to the ProteomeXchange Consortium via the PRIDE partner repository with the dataset identifier PXD051410 and 10.6019/PXD051410.

## Abbreviations

DAPI: 4′,6-Diamidine-2′-phenylindole dihydrochloride
FCCP: Carbonyl cyanide 4-(trifluoromethoxy)phenylhydrazone
GO: Gene ontology
HIF1α: Hypoxia-inducible factor 1 alpha
mtDNA/nDNA: Ratio of mitochondrial to nuclear DNA copy number
NV: Neovascularization
OIR: Oxygen-induced retinopathy
OCR: Oxygen consumption rate
P: Postnatal day
PSD95: Postsynaptic density protein 95
VO: Vaso-obliteration
