# Correction: Mitochondrial control of hypoxia-induced pathological retinal angiogenesis

**DOI:** 10.1007/s10456-024-09952-6

**Published:** 2024-10-19

**Authors:** Hitomi Yagi, Myriam Boeck, Shen Nian, Katherine Neilsen, Chaomei Wang, Jeff Lee, Yan Zeng, Matthew Grumbine, Ian R. Sweet, Taku Kasai, Kazuno Negishi, Sasha A. Singh, Masanori Aikawa, Ann Hellström, Lois E. H. Smith, Zhongjie Fu

**Affiliations:** 1 Department of Ophthalmology, Boston Children’s Hospital, Harvard Medical School, 3 Blackfan Circle, CLS 18, Boston, MA 02115 USA; 2 Department of Ophthalmology, Keio University School of Medicine, Tokyo, 160-8582 Japan; 3 Eye Center, Medical Center, Faculty of Medicine, University of Freiburg, 79106 Freiburg, Germany; 4 Department of Pathology, Xi’an Medical University, Xi’an, 710021 Shaanxi Province China; 5 EnTox Sciences, Inc., Mercer Island, WA 98040 USA; 6 University of Washington Medicine Diabetes Institute, University of Washington, Seattle, WA 98109 USA; 7 Center for Interdisciplinary Cardiovascular Sciences, Division of Cardiovascular Medicine, Department of Medicine, Brigham Women’s Hospital, Harvard Medical School, Boston, MA 02115 USA; 8 Center for Excellence in Vascular Biology, Division of Cardiovascular Medicine, Brigham and Women’s Hospital, Harvard Medical School, Boston, MA 02115 USA; 9 Channing Division of Network Medicine, Department of Medicine, Brigham Women’s Hospital, Harvard Medical School, Boston, MA 02115 USA; 10 The Sahlgrenska Centre for Pediatric Ophthalmology Research, Department of Clinical Neuroscience, Institute of Neuroscience and Physiology, Sahlgrenska Academy, University of Gothenburg, 405 30 Gothenburg, Sweden

**Correction to: Angiogenesis** 10.1007/s10456-024-09940-w

The wrong Supplementary file was originally published with this article; it has now been replaced with the correct file.

The original article has been corrected.

## Supplementary Information

Below is the link to the electronic supplementary material.

Supplementary file1 (DOCX 50 KB)

