# Supplementary material for: Mitochondrial control of hypoxia-induced pathological retinal angiogenesis
Source: Angiogenesis. 2024 Aug 3;27(4):691–9. doi: 10.1007/s10456-024-09940-w (PMC11564381; doi:10.1007/s10456-024-09940-w)
Supplement: Supplementary file 1 — Supplementary file1 (DOCX 51 KB) [file 10456_2024_9940_MOESM1_ESM.docx]

Journal: Angiogenesis

**Mitochondrial control of hypoxia-induced pathological retinal angiogenesis**

Hitomi Yagi^1,2,*^, Myriam Boeck^1,3,*^, Shen Nian^1,4^, Katherine Neilsen^1^, Chaomei Wang^1^, Jeff Lee^1^, Yan Zeng^1^, Matthew Grumbine^5^, Ian R Sweet^6^, Taku Kasai^7^, Kazuno Negishi^2^, Sasha A. Singh^7^, Masanori Aikawa^7,8,9^, Ann Hellström^10^, Lois EH Smith^1,#^, Zhongjie Fu^1,#^

^1^Department of Ophthalmology, Boston Children’s Hospital, Harvard Medical School, Boston, MA 02115, USA.

^2^Department of Ophthalmology, Keio University School of Medicine, 160-8582 Tokyo, Japan.

^3^Eye Center, Medical Center, Faculty of Medicine, University of Freiburg, 79106 Freiburg, Germany.

^4^Department of Pathology, Xi’an Medical University, Xi’an, 710021, Shaanxi Province, China.

^5^EnTox Sciences, Inc., Mercer Island, WA 98040, USA.

^6^University of Washington Medicine Diabetes Institute, University of Washington, Seattle, WA 98109, USA.

^7^Center for Interdisciplinary Cardiovascular Sciences, Division of Cardiovascular Medicine, Department of Medicine, Brigham Women's Hospital, Harvard Medical School, Boston, MA 02115, USA.

^8^Center for Excellence in Vascular Biology, Division of Cardiovascular Medicine, Brigham and Women’s Hospital, Harvard Medical School, Boston, MA 02115, USA.

^9^Channing Division of Network Medicine, Department of Medicine, Brigham Women's Hospital, Harvard Medical School, Boston, MA 02115, USA.

^10^The Sahlgrenska Centre for Pediatric Ophthalmology Research, Department of Clinical Neuroscience, Institute of Neuroscience and Physiology, Sahlgrenska Academy, University of Gothenburg, Gothenburg 405 30, Sweden.

*These authors contributed equally.

^#^Corresponding authors:

Zhongjie Fu, PhD (email: [zhongjie.fu@childrens.harvard.edu](mailto:jgidda@lsuhsc.edu)), Department of Ophthalmology, Boston Children’s Hospital, Harvard Medical School, 3 Blackfan Circle, CLS 18, Boston, MA 02115, USA. Tel: (+1) 617 919 2534

Lois EH Smith, MD, PhD (email: lois.smith@childrens.harvard.edu), Department of Ophthalmology, Boston Children’s Hospital, Harvard Medical School, 3 Blackfan Circle, CLS 18, Boston, MA 02115, USA. Tel: (+1) 617 919 2529

**Supplemental Materials**

**Supplemental Methods**

**SUPPLEMENTAL METHODS**

**Mouse model of oxygen-induced retinopathy (OIR)**

All mouse experiments followed the ARVO Statement for Use of Animals in Ophthalmic Vision and Research and the ethical principles established by the National Institutes of Health Guide for the Care and Use of Laboratory Animals (NIH Publications No. 8523, revised 2011). The work was approved and in accordance with Boston Children’s Hospital’s Institutional Animal Care and Use Committee (IACUC) protocol No. 00001619.

C57BL/6J mouse pups and their nursing dam were exposed to 75% O_2_ (ProOx Model 110, Biospherix) from postnatal day (P)7-P12 and returned to room air (21% O_2_) at P12, resulting in hyperoxia-induced central retinal vaso-obliteration. Relative hypoxia-induced midperipheral retinal neovascularization begins to form from P14 and peaks at P17 [1] (**Fig. 1a**). Control mice were maintained in room air. For BaroFuse analysis of P17 OIR vs. normal control mice, one litter was split into two cages, each surrogated with a nursing dam.

For *in vivo* testing, sodium pyruvate (50 μg/g body weight, P2256, Sigma-Aldrich) or vehicle control (phosphate buffered saline, PBS) was intraperitoneally administered to littermate mouse pups daily from P12-P14 (prior to onset of neovessel formation) or P14-P16 (during neovessel formation). Sodium pyruvate at a lower (10 μg/g) and higher (70 μg/g) dose from P14-P16 was also tested in OIR. Retinas were collected at P17 and stained with isolectin GS-IB4 (vessel marker, Invitrogen, I21413). Images were taken using a Zeiss confocal microscope. Retinal vascular pathology was quantified with Image J plugin SWIFT_NV [2,3].

**Proteomics analysis using tandem mass tag (TMT) mass spectrometry**

For TMT mass spectrometry, retinas were isolated at P17 from OIR and normal control mice (n=6 mice/group). Total proteome analysis was conducted at the Thermo Fisher Center for Multiplexed Proteomics at Harvard Medical School. The mass spectrometry proteomics data have been deposited to the ProteomeXchange Consortium via the PRIDE [4] partner repository with the dataset identifier PXD051410 and 10.6019/PXD051410. There were 18 samples in the TMT dataset. Here, we report on 12 samples, and 6 samples will be reported in a separate study.

*Sample preparation for mass spectrometry*: Samples for protein analysis were prepared as described [5,6]. Proteins were extracted with buffer containing 200 mM 4-(2-Hydroxyethyl)-1-piperazinepropanesulfonic acid (EPPS) (E9502, Sigma-Aldrich) pH 8.5, 8 M urea, and protease inhibitors. Following lysis, each sample was reduced with 5 mM Tris(2-carboxyethyl)phosphine hydrochloride (TCEP) (77720, Thermo Fisher Scientific). Cysteine residues were alkylated using 10 mM iodoacetamide for 20 min at room temperature (RT) in the dark. Excess iodoacetamide was quenched with 10 mM dithiothreitol (DTT). 100 µg of each proteome were precipitated and re-solubilized in 200 mM EPPS pH 8.5. Samples were digested with the endoproteinase Lys-C (1:50) overnight at RT and subsequently with trypsin (1:100) for 6 hr at 37°C. Anhydrous acetonitrile was added to each sample to achieve a final concentration of 33% acetonitrile. 50 µg of peptides from each sample were labeled with TMTPro reagents (A44520, Thermo Fisher Scientific) for 2 hr at RT. Labeling reactions were quenched with 0.5% hydroxylamine and acidified with formic acid. Acidified peptides were combined and desalted by Sep-Pak (WAT054960, Waters).

*Basic pH reversed-phase separation (BPRP):* TMT-labeled peptides were solubilized in 5% acetonitrile/10 mM ammonium bicarbonate pH 8.0 and ~300 µg of TMT-labeled peptides were separated by an Agilent 300 Extend C18 column (3.5 µm particles, 4.6 mm ID, and 250 mm in length). An Agilent 1260 binary pump coupled with a photodiode array (PDA) detector (056126, Thermo Scientific) was used to separate the peptides. A 45 min linear gradient from 10% to 40% acetonitrile in 10 mM ammonium bicarbonate pH 8.0 (flow rate of 0.6 mL/min) separated the peptide mixtures into a total of 96 fractions (36 s) which were consolidated into 24 samples in a checkerboard fashion and vacuum-dried to completion. Each sample was desalted via Stage Tips and re-dissolved in 5% formic acid (FA)/ 5% ACN for mass spectrometry.

*Liquid chromatography separation and tandem mass spectrometry (LC-MS3):* Spectra were collected on an Orbitrap Fusion Lumos mass spectrometer (Thermo Fisher Scientific) coupled to a Proxeon EASY-nLC 1000 LC pump (Thermo Fisher Scientific). Fractionated peptides were separated using a 180 min gradient at 600 nL/min on a 35 cm column (i.d. 100 μm, Accucore, 2.6 μm, 150 Å) packed in-house. MS1 data were collected in the Orbitrap (120,000 resolution; maximum injection time 50 ms; automatic gain control (AGC) 10 × 105). Charge states between 2 and 5 were required for MS2 analysis, and a 180 s dynamic exclusion window was used. Top 10 MS2 scans were performed in the ion trap with collision induced dissociation (CID) fragmentation (isolation window 0.5 Da; Rapid; normalized collision energy (NCE) 35%; maximum injection time 50 ms; AGC 1.2 × 104). An on-line real-time search algorithm (Orbiter) was used to trigger MS3 scans for quantification [7]. MS3 scans were collected in the Orbitrap using a resolution of 50,000, NCE of 65%, maximum injection time of 200 ms, and AGC of 3.0 × 105. The close out was set at 2 peptides per protein per fraction [7].

*Data analysis:* Raw files were converted to mzXML, and monoisotopic peaks were re-assigned using Monocle [8]. Searches were performed using the Comet search algorithm against a mouse database downloaded from Uniprot in May 2021. The database contains 63914 forward sequences. However, to estimate False Discovery Rate (FDR), we also included all the reverse sequences for each of those 63,914 sequences. Therefore, the total number of entries is 127,828 (63,914x2). We used a 50 ppm precursor ion tolerance, 1.0005 fragment ion tolerance, and 0.4 fragment bin offset for MS2 scans collected in the ion trap. TMTpro on lysine residues and peptide N-termini (+304.2071 Da) and carbamidomethylation of cysteine residues (+57.0215 Da) were set as static modifications, while oxidation of methionine residues (+15.9949 Da) was set as a variable modification.

Each run was filtered separately to 1% FDR on the peptide-spectrum match (PSM) level. Then, proteins were filtered to the target 1% FDR level across the entire combined data set. For reporter ion quantification, a 0.003 Da window around the theoretical mass-to-charge ratio (m/z) of each reporter ion was scanned, and the most intense m/z was used. Reporter ion intensities were adjusted to correct for isotopic impurities of the different TMTpro reagents according to manufacturer specifications. Peptides were filtered to include only those with a summed signal-to-noise (S/N) ≥ 180 across all TMT channels. The S/N measurements of peptides assigned to each protein were summed (for a given protein).

Data were further analyzed using the statistical software, Qlucore (Qlucore, Sweden, version 3.5). We performed a two-group comparison (OIR vs. normal control) using the log-transformed protein group means, the student’s t-test for each protein’s comparison (P value), and the Benjamini-Hochberg procedure to calculate the FDR adjusted P value (q value). Proteins with significantly enriched statistically increased or decreased abundance with q<0.05 were inputted into the Enrichr database [9-11] for gene ontology (GO) term analysis of biological processes. Enrichr provided P values computed from the Fisher’s exact test, and adjusted P value computed using the Benjamini-Hochberg method for correction for multiple hypotheses testing. Pathways were considered as significant with adjusted P<0.05. Principal component analysis (PCA) was performed on unfiltered proteome (q=1).

**Immunohistochemistry (IHC)**

IHC was performed as described [12]. Briefly, mouse eyes were vertically sectioned using a Leica CM3050 S Cryostat (Leica). Sections with retina and the optic nerve were treated with ice-cold methanol and 0.1% Triton PBS subsequently. Retinal sections were blocked with 3% bovine serum albumin (BSA) for 1 hr at RT and stained with primary antibodies against synaptophysin (1:200, MAB368, Millipore) and postsynaptic density protein 95 (PSD95) (1:200, 75-028, UC Davis/NIH NeuroMab Facility) overnight at 4°C. Corresponding fluorescent secondary antibodies were then incubated for 1 hr at RT, and sections were covered in mounting medium with 4′,6-diamidine-2′-phenylindole dihydrochloride (DAPI for cell nuclei, H‑1200, Vector Laboratories). Images were taken using a Zeiss confocal microscope at 200X magnification.

**Mitochondrial DNA copy number**

The mitochondrial DNA copy number ratio (mtDNA/nDNA) was estimated using cDNA from mitochondrial RNA which was divided by cDNA from nuclear RNA. Total RNA, including mitochondrial and nuclear RNA, was extracted from P12, P14, and P17 OIR and normal control retinas using PureLink™ RNA Mini Kit (12183018A, Invitrogen). The cDNA was synthesized with iScript Reverse Transcription Supermix (1708841, Bio-Rad). The mtDNA/nDNA ratio was estimated by applying 5 ng cDNA samples to the mouse mitochondrial DNA copy number kit (MCN3, Detroit R&D). The fold change was calculated by using ΔΔ threshold cycle method and normalizing to the control group.

**BaroFuse analysis**

Oxygen-consumption rate (OCR), reflecting mitochondrial respiration (potassium cyanide (KCN) -sensitive OCR), was measured in P12, P14, and P17 *ex vivo* retinas using the multichannel microfluidics device BaroFuse (BF-OXY-FC-8, EnTox Sciences, Inc.) [13-16]. In the BaroFuse apparatus, two media reservoirs each perfuse four tissue chambers. After configuring the instrument and achieving equilibrium between the medium and the gas in the reservoir’s headspace (21% O_2_, 5% CO_2_, balance N_2_), tissue chambers were loaded with a single retina each, followed by equilibration for at least 90 min at approximately 25-35 μL/min to achieve OCR baseline stabilization. For each side of the BaroFuse three out of four channels were loaded with a retina each while one remained as a no tissue control enabling downstream tissue data correction for inflow oxygen changes over time. Where possible, to control for animal-to-animal variability each retina from a single mouse was positioned in opposite media reservoirs. For experiments comparing OCR of OIR vs. control retinas, 70 mL of the following buffer were added to each reservoir: HEPES-buffered Krebs-Ringer Solution (J67795, Thermo Fisher Scientific) with 5 mM glucose and 0.1% BSA. After baseline stabilization, the ATP synthase inhibitor oligomycin A (11342, Cayman Chemicals, 30 μM from a 15 mM stock in dimethylsulfoxide (DMSO)) was added to both sides of the system, followed 60 min later by adding the mitochondrial uncoupler carbonyl cyanide 4‑(trifluoromethoxy)phenylhydrazone (FCCP) (C2920, Sigma-Aldrich, 1 μM from a 1 mM stock in DMSO). For experiments assessing the ability of pyruvate to sustain OCR upon glucose deprivation, one reservoir was filled with 70 mL HEPES-buffered Krebs-Ringer Solution (J67795, Thermo Fisher Scientific) containing 10 mM pyruvate (11360-070, Gibco), 1 mM glucose, and 0.1% BSA. For the other reservoir’s medium, pyruvate volume was replaced by double-distilled water. After baseline stabilization, the glucose transporter inhibitor BAY-876 (HY-100017, MedChemExpress, 20 μM from a 10 mM stock in DMSO) was injected into both reservoirs. At the end of every experiment, an injection of the complex IV inhibitor KCN (207810, Sigma-Aldrich, 3 mM from a 3 M stock in double-distilled water) was applied to inhibit mitochondrial respiration and determine the inflow O_2_ signal of each channel. OCR was calculated and visualized using the Barofuse Data Processor (v2.20.0) as previously described [17]. Results from at least 2 independent experiments were shown (exception P14: 1 experiment).

**Statistics**

Statistics are described in the figure legends. In general, normality (quantile-quantile (QQ) plot) and F-test (for variance) were conducted first, followed by the according parametric unpaired t-test (or Welch’s t-test) or the non-parametric Mann-Whitney test to compare groups (Prism v9.0; GraphPad Software, Inc.). P<0.05 was considered statistically significant.

**REFERENCES**

1. Smith LE, Wesolowski E, McLellan A, Kostyk SK, D'Amato R, Sullivan R, D'Amore PA (1994) Oxygen-induced retinopathy in the mouse. Invest Ophthalmol Vis Sci 35 (1):101-111

2. Connor KM, Krah NM, Dennison RJ, Aderman CM, Chen J, Guerin KI, Sapieha P, Stahl A, Willett KL, Smith LE (2009) Quantification of oxygen-induced retinopathy in the mouse: a model of vessel loss, vessel regrowth and pathological angiogenesis. Nature protocols 4 (11):1565-1573. doi:10.1038/nprot.2009.187

3. Stahl A, Connor KM, Sapieha P, Willett KL, Krah NM, Dennison RJ, Chen J, Guerin KI, Smith LE (2009) Computer-aided quantification of retinal neovascularization. Angiogenesis 12 (3):297-301. doi:10.1007/s10456-009-9155-3

4. Perez-Riverol Y, Bai J, Bandla C, Garcia-Seisdedos D, Hewapathirana S, Kamatchinathan S, Kundu DJ, Prakash A, Frericks-Zipper A, Eisenacher M, Walzer M, Wang S, Brazma A, Vizcaino JA (2022) The PRIDE database resources in 2022: a hub for mass spectrometry-based proteomics evidences. Nucleic Acids Res 50 (D1):D543-D552. doi:10.1093/nar/gkab1038

5. Navarrete-Perea J, Yu Q, Gygi SP, Paulo JA (2018) Streamlined Tandem Mass Tag (SL-TMT) Protocol: An Efficient Strategy for Quantitative (Phospho)proteome Profiling Using Tandem Mass Tag-Synchronous Precursor Selection-MS3. Journal of proteome research 17 (6):2226-2236. doi:10.1021/acs.jproteome.8b00217

6. Li J, Cai Z, Bomgarden RD, Pike I, Kuhn K, Rogers JC, Roberts TM, Gygi SP, Paulo JA (2021) TMTpro-18plex: The Expanded and Complete Set of TMTpro Reagents for Sample Multiplexing. Journal of proteome research 20 (5):2964-2972. doi:10.1021/acs.jproteome.1c00168

7. Schweppe DK, Eng JK, Yu Q, Bailey D, Rad R, Navarrete-Perea J, Huttlin EL, Erickson BK, Paulo JA, Gygi SP (2020) Full-Featured, Real-Time Database Searching Platform Enables Fast and Accurate Multiplexed Quantitative Proteomics. Journal of proteome research 19 (5):2026-2034. doi:10.1021/acs.jproteome.9b00860

8. Rad R, Li J, Mintseris J, O'Connell J, Gygi SP, Schweppe DK (2021) Improved Monoisotopic Mass Estimation for Deeper Proteome Coverage. Journal of proteome research 20 (1):591-598. doi:10.1021/acs.jproteome.0c00563

9. Chen EY, Tan CM, Kou Y, Duan Q, Wang Z, Meirelles GV, Clark NR, Ma'ayan A (2013) Enrichr: interactive and collaborative HTML5 gene list enrichment analysis tool. BMC Bioinformatics 14:128. doi:10.1186/1471-2105-14-128

10. Kuleshov MV, Jones MR, Rouillard AD, Fernandez NF, Duan Q, Wang Z, Koplev S, Jenkins SL, Jagodnik KM, Lachmann A, McDermott MG, Monteiro CD, Gundersen GW, Ma'ayan A (2016) Enrichr: a comprehensive gene set enrichment analysis web server 2016 update. Nucleic Acids Res 44 (W1):W90-97. doi:10.1093/nar/gkw377

11. Xie Z, Bailey A, Kuleshov MV, Clarke DJB, Evangelista JE, Jenkins SL, Lachmann A, Wojciechowicz ML, Kropiwnicki E, Jagodnik KM, Jeon M, Ma'ayan A (2021) Gene Set Knowledge Discovery with Enrichr. Curr Protoc 1 (3):e90. doi:10.1002/cpz1.90

12. Fu Z, Qiu C, Cagnone G, Tomita Y, Huang S, Cakir B, Kotoda Y, Allen W, Bull E, Akula JD, Joyal JS, Hellstrom A, Talukdar S, Smith LEH (2021) Retinal glial remodeling by FGF21 preserves retinal function during photoreceptor degeneration. iScience 24 (4):102376. doi:10.1016/j.isci.2021.102376

13. Kamat V, Robbings BM, Jung SR, Kelly J, Hurley JB, Bube KP, Sweet IR (2021) Fluidics system for resolving concentration-dependent effects of dissolved gases on tissue metabolism. Elife 10. doi:10.7554/eLife.66716

14. Rountree A, Karkamkar A, Khalil G, Folch A, Cook DL, Sweet IR (2016) BaroFuse, a novel pressure-driven, adjustable-throughput perfusion system for tissue maintenance and assessment. Heliyon 2 (12):e00210. doi:10.1016/j.heliyon.2016.e00210

15. Tsantilas KA, Cleghorn WM, Bisbach CM, Whitson JA, Hass DT, Robbings BM, Sadilek M, Linton JD, Rountree AM, Valencia AP, Sweetwyne MT, Campbell MD, Zhang H, Jankowski CSR, Sweet IR, Marcinek DJ, Rabinovitch PS, Hurley JB (2021) An Analysis of Metabolic Changes in the Retina and Retinal Pigment Epithelium of Aging Mice. Invest Ophthalmol Vis Sci 62 (14):20. doi:10.1167/iovs.62.14.20

16. Bisbach CM, Hass DT, Robbings BM, Rountree AM, Sadilek M, Sweet IR, Hurley JB (2020) Succinate Can Shuttle Reducing Power from the Hypoxic Retina to the O2-Rich Pigment Epithelium. Cell reports 31 (5):107606. doi:10.1016/j.celrep.2020.107606

17. Kamat V, Grumbine MK, Bao K, Mokate K, Khalil G, Cook D, Clearwater B, Hirst R, Harman J, Boeck M, Fu Z, Smith LEH, Goswami M, Wubben TJ, Walker EM, Zhu J, Soleimanpour SA, Scarlett JM, Robbings BM, Hass D, Hurley JB, Sweet IR (2023) A versatile pumpless multi-channel fluidics system for maintenance and real-time functional assessment of tissue and cells. Cell Rep Methods 3 (11):100642. doi:10.1016/j.crmeth.2023.100642
